# Supplementary material for: Management practices and their relation to success of Polish SMEs: The empirical verification
Source: PLoS One. 2021 Nov 15;16(11):e0259892. doi: 10.1371/journal.pone.0259892 (PMC8592451; doi:10.1371/journal.pone.0259892)
Supplement: Attachment — Submitted filename: rebuttal_letter_corr (1).docx [file pone.0259892.s001.docx]

**REBUTTAL LETTER**

Reviewer #1

1. We have added information in the lines in part 1 – Introduction referring to the research on the directly studied issue of the relationship between management practices and the success of SMEs. We have cited research carried out under the aegis of supranational institutions, e.g. the European Parliament, at the national level, e.g. in Germany (Broszeit et al. 2016) or Great Britain (Forth, Bryson 2018), and by a state agency in Poland (eds. Skowrońska, Tarnawa 2021), and even at the regional level (see e.g. Kuchiński, Prymas 2019). On this basis, the existing research gap was also determined, which results from the fact that in post-socialist economies, comprehensive studies of management practices in SMEs, and in particular their impact on their success, have not been carried out so far, unlike in highly developed countries. These countries, however, as stated, play an increasingly important role in international economic exchange.

2. In the Originality/Value section, we have added a statement regarding research value. Their value results mainly from the fact that the study is the first in post-socialist economies to attempt a comprehensive identification of those practices of managing SMEs which are related to better economic results.

3. The revised version of the third part, Results, contains a detailed description of both the data acquisition process and its selection for the purposes of performing the analyzes contained in the article. The structure of the surveyed enterprises has also been characterized in terms of industry and size.

4. Also in the third part, Results, we have defined the SMEs studied in the article. According to the classification adopted in Poland, small enterprises employ from 10 to 49 people, and medium-sized enterprises – from 50 to 249.

5. It can be assumed that the Polish economy shows some similarities to other economies of the post-socialist countries of Central Europe, in particular the Czech Republic and Hungary, and to a lesser extent the Baltic countries or Ukraine. Characteristic for these countries may be some overestimation of technical and measurable aspects of management and underestimation of qualitative aspects resulting from backwardness in the area of professional management.

6. We have corrected citation errors throughout the article.

7. The statement that larger enterprises grow faster was made on the basis of research conducted on the same data set based on the SEM analysis (structural equations) as part of the “Determinants of the entrepreneurship development in the SMEs sector” project and published and available on the website of the Central Statistical Office: <https://stat.gov.pl/files/gfx/portalinformacyjny/pl/defaultstronaopisowa/6155/1/1/raport_rozwoj_przedsiebiorczosci_w_sektorze_msp.pdf>. These are therefore empirically confirmed conclusions. The review concluded that the conclusion was debatable and that small high-tech enterprises might contradict it. However, in the total number of enterprises surveyed, the share of such enterprises is small, simple small business dominates, so the statistical trace of such a process is blurred. It can therefore be concluded that statistically, in the SME group, growth precedes development and the greater the growth, the greater the chance for development.

8. The selection of management practices potentially determining the success of SMEs in Poland was carried out as a result of an analysis of previous research by other authors, focus studies with entrepreneurs carried out as part of the “Determinants of the entrepreneurship development in the SME sector” project and the corresponding author's own research. It is not the case that only the entry in lines 205–206 applies to it, as part of the article from line 64 to 130 is devoted to it. It includes not only citations of our own works, which the review suggested, but also numerous citations of research carried out by other authors. We have added studies such as Kohli & Jaworski 1990 and Davidsson 1989 in the corrected version; research carried out in the Eastern bloc, which also includes Poland, e.g. Grigore 2008; and a citation to the block of practices related to information technology (Levy et al. 2001)

9. We have edited methodological information from lines 179, 206, 238 and others, in which they appeared, so that they don’t duplicate in several places in the text.

10. The choice of an algorithm for grouping objects is strictly dependent on the measurement scale of the variables that describe these objects. In the presented work, all variables are expressed only on the ordinal scale. The ordinal character of the data required the use of an appropriate distance measure. In the study, the analysis of the similarities of enterprises is based on the GDM2 distance, which is dedicated to this type of scale.

The popular k-means algorithm is dedicated to grouping objects described by variables measured on a strong scale, i.e. continuous variables. Hence, in our case, it could not be used.

11. We have corrected the error.

12. We have added a number of literature items to deal directly with the relationship between SME management practices and their market success, which can be verified on the basis of the references and in the Introduction section.

13. The limitations of the study have been described in the third part, Results, and are related to the procedure of selecting enterprises for the study. Originally, the study was planned as a complete study. The selected sample is a result of missing responses at the first stage of data collection and eliminated responses with missing data at the second stage. Still, it can be defended by using the argument that it exceeds the minimum sample size by more than 7 times.

Reviewer #2

1. We agree entirely. It was a mental shortcut. The purpose of this article has been edited wherever it appears.

2. We have supplemented numerous literature items and added items related directly to the studied issue.

3. We have corrected the technical errors.

4. We have described the process of selecting enterprises for the study in detail in the third section, Results.

5. Also in the third section, Results, we have presented in detail the structure of the surveyed enterprises in terms of industry and size. We have stated that the survey was nationwide (Poland) and that enterprises came from all large administrative units (from each of the 16 voivodeships).

6. We have added sources for the tables.

7. No answer was required due to the acceptance of the quality of statistical analyzes.

8. No response was required due to the acceptance of the level of clarity and content-related value of the conclusions.

9. We believe that the conclusions will not change taking into account the industry structure and size of the surveyed companies.
